# Evidence of horizontal gene transfer by transposase gene analyses in *Fervidobacterium* species

**DOI:** 10.1371/journal.pone.0173961

**Published:** 2017-04-20

**Authors:** Alba Cuecas, Wirojne Kanoksilapatham, Juan M. Gonzalez

**Affiliations:** 1 Institute of Natural Resources and Agrobiology, Spanish Council for Research, IRNAS-CSIC, Avda. Rena Mercedes 10, Sevilla, Spain; 2 Department of Microbiology, Faculty of Science, Silpakorn University, Nakhon Pathom, Thailand; National Renewable Energy Laboratory, UNITED STATES

## Abstract

Horizontal Gene Transfer (HGT) plays an important role in the physiology and evolution of microorganisms above all thermophilic prokaryotes. Some members of the Phylum Thermotogae (i.e., *Thermotoga* spp.) have been reported to present genomes constituted by a mosaic of genes from a variety of origins. This study presents a novel approach to search on the potential plasticity of *Fervidobacterium* genomes using putative transposase-encoding genes as the target of analysis. Transposases are key proteins involved in genomic DNA rearrangements. A comprehensive comparative analysis, including phylogeny, non-metric multidimensional scaling analysis of tetranucleotide frequencies, repetitive flanking sequences and divergence estimates, was performed on the transposase genes detected in four *Fervidobacterium* genomes: *F*. *nodosum*, *F*. *pennivorans*, *F*. *islandicum* and a new isolate (*Fervidobacterium* sp. FC2004). Transposase sequences were classified in different groups by their degree of similarity. The different methods used in this study pointed that over half of the transposase genes represented putative HGT events with closest relative sequences within the phylum Firmicutes, being *Caldicellulosiruptor* the genus showing highest gene sequence proximity. These results confirmed a direct evolutionary relationship through HGT between specific *Fervidobacterium* species and thermophilic Firmicutes leading to potential gene sequence and functionality sharing to thrive under similar environmental conditions. Transposase-encoding genes represent suitable targets to approach the plasticity and potential mosaicism of bacterial genomes.

## Introduction

The microbial world presents an astonishing high diversity [1, 2]. Natural habitats contain complex microbial communities where different microorganisms interact with the environment representing a driving force to get adapted to the available conditions. The microbial cells live in communities and they continuously interact with others both within and between species [3]. This interactive scenario generates opportunities to exchange functional capabilities and accelerate the pace of evolutionary adaptation.

Horizontal gene transfer (HGT) represents the exchange of DNA between species and, this is even more interesting when it occurs among scarcely related cells, for example, between different phyla. HGT events occur frequently in the prokaryotic world and they are thought to have enormous relevance in the evolution of prokaryotes [4, 5, 6]. However, the mechanisms governing these events are poorly understood. Phylogenetic studies are providing valuable information on potential DNA mobility events during microbial evolution. In this respect, the involvement of mobile genetic elements is a major factor involved in the genomic evolution of prokaryotes [6, 7].

The activities of different mobile genetic elements, both within a single genome and across genomes of different microorganisms, have serious impacts on genome structure and function. For instance, mobile genetic elements are able to generate transpositions of DNA fragments and as a result gene inactivation/activation and gene deletions and insertions [8]. Among the mobile elements, the insertion sequences (IS) are considered the simplest autonomous transposition elements. These ISs are constituted by a transposase gene flanked up- and down-stream by repetitive flanking sequences. Consequently, transposase genes are expected to show genome rearrangements more frequently than other genes [9]. The mechanism of action of transposases consists of replicative and non-replicative (conservative) modes that facilitate movement of the transposition elements to a different location. As a consequence, gene disruption caused by the scission of an IS and its insertion into the genome are usually traced back to understand these evolutionary events [10]. The repetitive flanking sequences, or inverted repeat (IR) sequences, are targets of the transposases and are generally included in the transposed DNA. The involvement of transposases in the mobility of DNA within and likely between genomes [6, 7] suggests that these genes can be used as a proxy to better understand the potential of HGT on bacterial evolution and genome plasticity.

Extreme conditions, such as high temperatures, represent environments fostering rapid adaptation mechanisms. HGT has proven to represent a major mechanism allowing the adaptation of microorganisms to these environments [11, 12, 13]. For instance, the phylum Thermotogae, mostly represented by thermophiles, has been reported to present a high frequency of HGT events, such as some DNA fragments putatively coming from members of the Phylum Firmicutes and the Domain Archaea. This has been pointed out in the genome of *Thermotoga maritima* [6, 14], genomes of *Thermotoga* and *Thermosipho* species and *Fervidobacterium nodosum* [13, 15].

In this study, we analyzed transposase genes in *Fervidobacterium* genomes as potential targets to infer the putative occurrence of HGT events in this genus and their liaison to distantly and closely related taxa.

## Materials and methods

### Genomes, genes and phylogenies

The genomes of *F*. *nodosum* Rt17-B1 (NC_009718) [13, 16], *F*. *pennivorans* DSM9070 (NC_017095) [17], *F*. *islandicum* AW-1 (NZ_CP014334) [18, 19] and a new isolate [20], *Fervidobacterium thailandense* strain FC2004 (LWAF01000000) were used in this analysis. Annotated transposases in these four genomes and homology searching (using blast algorithms) [21] resulted in the detection of a number of transposases. They were classified based on their similarity to transposase families as proposed by Siguier et al. [8] at the ISFinder database (http://www-is.biotoul.fr). Blastp was used to determine the closest relatives to the transposase genes detected in *Fervidobacterium* genomes. Besides the closest relative sequences detected in GenBank, the closest sequences to those transposase families within the Phylum Thermotogae were also incorporated into the analyses as a comparative threshold for the detection of HGT events between different phyla. Sequence alignments were performed by ClustalW [22]. Phylogenetic trees based on the amino acid sequences encoded by the detected genes were constructed using MEGA [23] by the Neighbor-joining method with a bootstrap value of 1000.

### Multivariate analyses of tetranucleotide frequencies

Non-metric MultiDimensional Scaling (NMDS) analyses were performed to obtain graphical distributions of the transposase gene sequences corresponding to each transposase family detected in the *Fervidobacterium* genomes and their related genes. The frequencies of tetranucleotides [24] were used in NMDS analyses. NMDS plots were constructed using R with the Vegan Package [25].

### Repetitive flanking sequences

The conservation of insertion sequence endings were studied by searching through visual inspection the sequence alignments corresponding to related transposase genes plus additional 1000 nucleotides up- and down-stream. When present, the detection of the inverted repeats or palindromic sequences (depending on the transposase family) was guided by the information available at the ISFinder database. The percentage of identity between aligned, conserved endings and the distance to the annotated start or stop codons were noted.

### K-L divergence estimates

The Kullback-Leiber (K-L) divergence metric [26, 27] was used as an approach to estimate the divergence between related transposase genes clustered in a transposase family. K-L divergence (D_KL_) was calculated on tetranucleotide frequencies for pairwise comparisons as

$$D_{KL}\left(g\;//G\right)=\sum g\left(i\right)\text{ln}\left(g\left(i\right)/G\left(i\right)\right)$$ (1)

where g is the parameter used (e.g., frequency of a particular tetranucleotide, i) for the analyzed gene and G the same parameter for a reference transposase gene or whole genome.

## Results

### Classification of transposases in *Fervidobacterium* genomes

The number of putative transposase genes detected in the *Fervidobacterium* (Phylum Thermotogae) genomes ranged between 39 and 48 per genome (Table 1). These transposase genes were classified in six IS families (IS6, IS110, IS200/IS605, ISL3, IS3 and IS4) plus four undefined groups (types A, B, C and D) that were unrelated to transposase families listed in ISFinder [28]. In Table 1, ISCpe7-like transposases (IS6 family) were identified as the most frequent IS element family in the genomes of *Fervidobacterium*: *F*. *nodosum* Rt17-B1 (23 copies), *F*. *pennivorans* DSM 9078 (24 copies), *Fervidobacterium* sp. FC2004 (12 copies) and *F*. *islandicum* AW-1 (34 copies). Based on their degree of similarity and phylogenetic relationships, the IS110 (IS110_I–IS110_III), IS200 (IS200_I-IS200_III), IS605 (IS605_I–IS605_III) and IS3 (IS3_I—IS3_III) were further classified into three subgroups. Evidences of HGT *Fervidobacterium* genomes that might have had occurred across phyla (i.e., other than Thermotoga) were characterized. The results revealed that ISCpe7, IS110, IS605_I, IS605_II, ISL3, IS3_II, IS3_III and the types B, C, and D might be actively involved in the HGT events rendered by other species belonging to other phyla. The IS605_I, ISL3, type C and type D were chosen as four representative models which were described below.

**Table 1:** Classification of insertion sequences detected in four *Fervidobacterium* genomes.

IS Family	Transposase groups	*F*. *nodosum* Rt17-B1	*F*. *pennivorans* DSM 9078	*Fervidobacterium* sp. FC 2004	*F*. *islandicum* AW-1	HGT^1^
IS6	ISCpe7-like	23	24	12	34	y
IS110	IS116/IS110/IS902-like					
	IS110_I	6				y
	IS110_II	2	1		1	n
	IS110_III	1	6			n
IS200/IS605	IS200-like					
	IS200_I			1		n
	IS200_II			3		n
	IS200_III				2	n
	IS605 OrfB-like					
	IS605_I	1				y
	IS605_II		1	6		y
	IS605_III			1	2	n
ISL3	IS204/IS1001/IS1096/IS1165-like	8				y
IS3	IS3/IS911-like					
	IS3_I	2				n
	IS3_II				2	y
	IS3_III				1	y
IS4	IS4-like			6		n
Type A	Type A		2			n
Type B	Type B		4			y
Type C	Type C		3		1	y
Type D	Type D			5		y
Total transposases		43	41	39	43	

^1^ Putative acquisition of these sequences through interphylum HGT. Yes, y; No, n.

### IS605_I transposases

**Fig 1:**
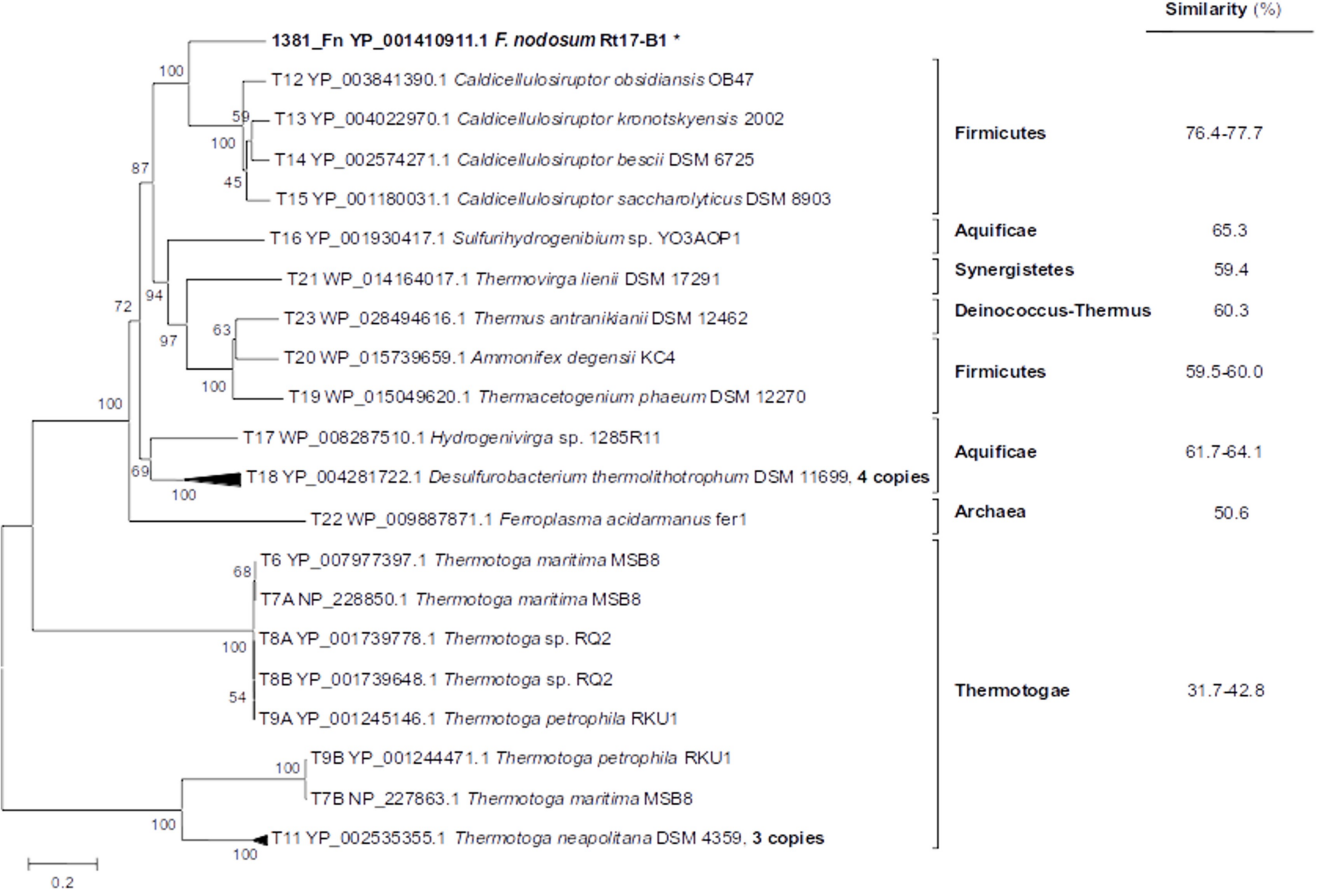
Phylogenetic relationship among *Fervidobacterium nodosum* and related gene sequences of the IS605_I transposase family and their closest relatives within the Phylum Thermotogae. The percentage of similarity related to the *F*. *nodosum* amino acidic sequence is shown on the right column.

Phylogenetic analysis using transposase sequences revealed a copy of IS605_I in *F*. *nodosum* which shares the highest similarity to a clade formed by *Caldicellulosiruptor* (Phylum Firmicutes; amino acid similarity 77%; Fig 1). Substantial transposase amino acid sequence similarities of 60–65% and 51% were observed in bacteria belonging to the phyla Aquificae, Synergistetes and Deinococcus-Thermus and a member of the Domain Archaea (*Ferroplasma acidarmanus*), respectively. In contrast, the closest Thermotogae representatives (*Thermotoga* spp.) for this transposase group formed a different cluster (Fig 1) showing much lower similarity (32–43%). Thermotogae and Firmicutes are too different phyla represented in phylogenetic dendrograms as independent low-branching bifurcations [29]. This suggests that the potential origin of these transposases in *F*. *nodosum* might have been the Phylum Firmicutes rather than the closest relatives in the Phylum *Thermotogae*.

**Fig 2:**
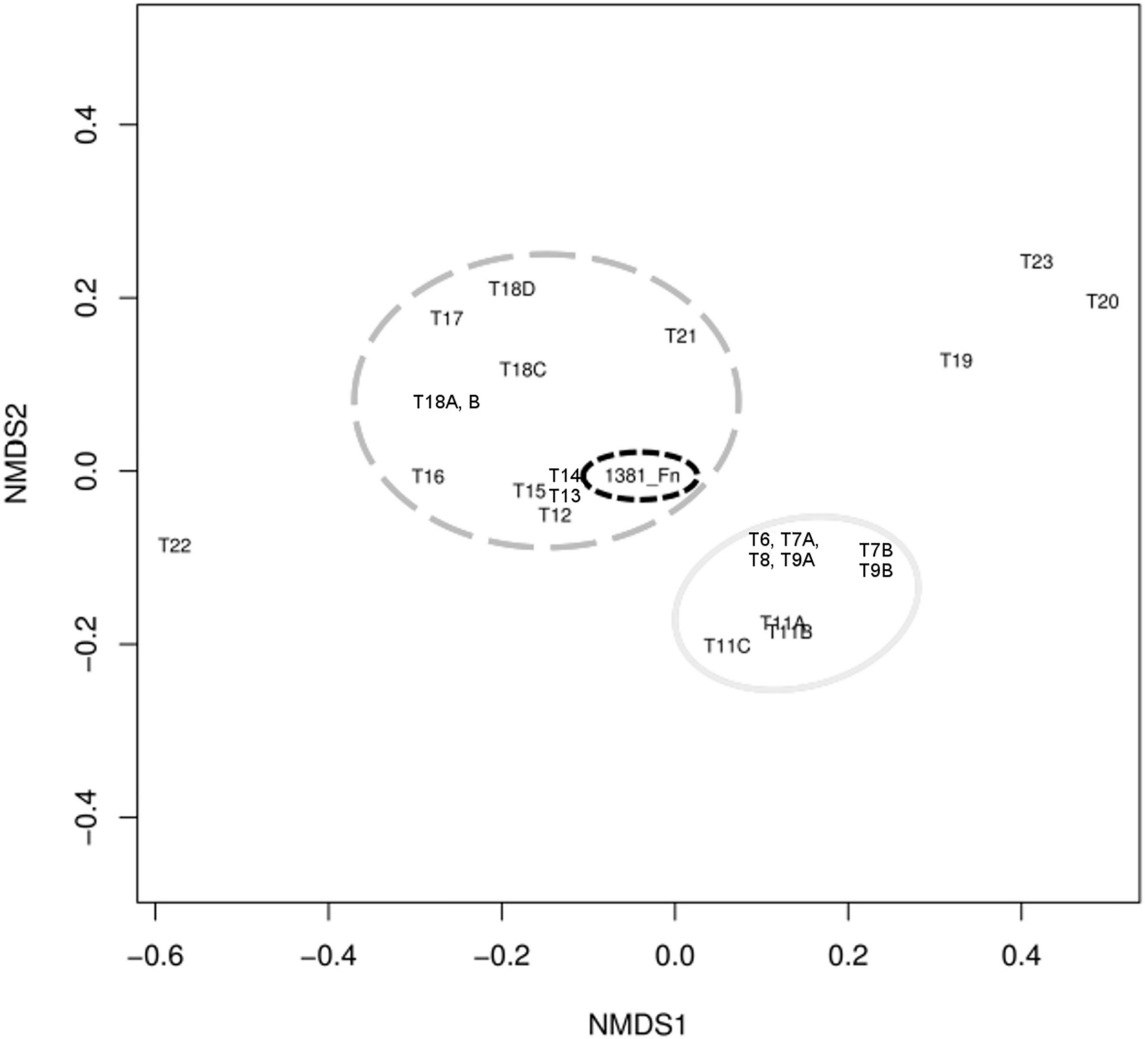
Non-metric MultiDimensional Scaling (NMDS) analysis of tetranucleotide frequencies for *F*. *nodosum* and related gene sequences of the IS_605_I transposase family. Identification of transposases follows the IDs used in the dendrogram for these sequences (Fig 1). Dashed black, *F*. *Nodosum*; dashed grey, diverse phyla related to the transposase in *F*. *Nodosum*; continuous grey, other Thermotogae.

NMDS analysis of tetranucleotide frequencies also showed a clustering of the IS605_I transposase from *F*. *nodosum* (1381_Fn) in the vicinity of the closest relatives (Fig 2) mainly from the genus *Caldicellulosiruptor* (Phylum Firmicutes). The results imply that the IS605 from *F*. *nodosum* is the closest relative to this type of transposition elements from *Caldicellulosiruptor*.

Analysis of palindromic IR sequences flanking the transposase gene sequences revealed that the left and the right IR sequences of *F*. *nodosum* show remarkably high nucleotide identity, 67–70% and 71–75%, to sequences from species of the genus *Caldicellulosiruptor* (Phylum Firmicutes), and to other Firmicutes, 55 and 60% to *Thermacetogenium phaeum* and 73 and 78% to *Ammonifex degensii*. Lower similarities to the left and right IR sequences from members belonging to the Phylum Aquificae (*Hydrogenivirga*, 42% and 57%, and *Desulfurobacterium*, 44–46% and 76–80%) were observed. In contrast, lowest similarities to the left and right IRs were obtained from members of the Phylum Thermotogae (42–43% and 55–60%). Besides, these IR sequences were highly conserved at the genus level and the high identity observed between these related sequences (i.e., *Fervidobacterium* and *Caldicellulosiruptor*) suggested interphyla relationships (Table 2).

**Table 2:** Repetitive flanking sequences detected in *F*. *nodosum* and related sequences within the IS605_I transposase family. The palindromic inverted repeats upstream (left end) and down-stream (right end) and their percentage of identity with respect to *F*. *nodosum* sequences are presented. Identification numbers correspond to those used in the figures for this transposase group.

ID	Accession No.	Organism	Left end	Distance^1^	Identity^1^	Distance^1^	Right end	Identity^1^
1381_Fn	YP_001410911	*Fervidobacterium nodosum* RT17-B1	TTACG–TAAATCTACCT–GAAAGGACATGTACTT–CAAGGTATTGGGCTGAAC	60		0	AGCA–GGAAGCCAAT–CCCTTA–A–AAG–GGG––TAGGAGGAGGTCAA	
T12	YP_003841390	*Caldicellulosiruptor obsidiansis* OB47	....AAA.G...AG..A–.....C....CCC...T..G..G....T.A....T	94	67.3	22	.––––....T..T..C.....C––––..–...AT.G............	72.7
T15	YP_001180031	*Caldicellulosiruptor saccharolyticus* DSM 8903	....AAAG....AG..A– .....C....C.C...T–.G..G....T.A....T	78	67.3	85	.CATG....T..T..C.....C––––..–...AT.G............	71.1
T13	YP_004022970	*Caldicellulosiruptor kronotskiensis* 2002	....AGAG....AG..A–.....C....CCC...–..G..G....T.A....T	94	68.6	22	..A––....T..T..C.....C––––..–...AT.G............	75.0
T14	YP_002574271	*Caldecellulosiruptor bescii DSM* 6725	....A–AG....AG..A–.....C....TCC...–..G..G....T.A....T	93	70.0	22	..A––....T..T..C.....C––..–...AT.G............	75.0
T20	YP_003239632	*Ammonifex degensii* KC4	.....AG.GGC.GC.GG–.....CC..––CT...TT..A.G––....A....A	57	55.7	1	..G––.......C.C–..T..C––––.A–...––.G...........C	73.1
T19	YP_006919201	*Thermacetogenium phaeum* DSM 12270	.....A.CCCC.GC.GG–.....CC..––CC...T.....G––....A....A	67	59.6	1	..A––.......C.C–.....T––––..–...––.G...........C	78.0
T17	WP_008287510	*Hydrogenivirga* sp. 128-5-R1-1	.....AA...A–....A–––G.ATG..CCCGT..TA..ACG––G.T.A....T	76	42.3	-21	....–......TCGGCTT...T––––.A–A.C––CGA.....T....C	57.1
T18D	YP_004281281	*Desulfurobacterium thermolithotrophum* DSM 11699	....AG......–CAGC–..G.AA..TCTGCT..T...A––GCA...A.A..T	55	46.1	-26	....–.......T.C–.A..–––.–...–T..––........T....C	80.4
T18C	YP_004281398	*Desulfurobacterium thermolithotrophum* DSM 11699	....AGA...C.GTTGC–..G.AAG.CCTGCT..TT..A––GCA...A....T	52	44.2	-20	....–.......CC.–.A...T––––..–T.A––GG......T....C	70.7
T18A	YP_004281722	*Desulfurobacterium thermolithotrophum* DSM 11699	....AGA...A..TTGC–..G.AAG.CCTGCT..TT..A––GCA...A.A..T	51	44.2	0	....–.......C.C–.A...T––––..–T..––.G......T....C	75.6
T18B	YP_004281103	*Desulfurobacterium thermolithotrophum* DSM 11699	....AGA...–..TTGC–..G.AAG.CCTGCT..TT..A––GCA...A.A..T	51	44.2	-20	....–.......T.C–.A...T––––..–T..––........T....C	78.0
T6	YP_007977397	*Thermotoga maritima* MSB8	...TCGA.T.AAAC.GCAA.G.A....CCATT..CT.TAAGG.G...A....T	127	43.3	0	...G–.......–..–GA...CT.T...–TCA––.G.T..––T–...C	60.4
T7A	NP_228850	*Thermotoga maritima* MSB8	...TCGA.T.AAAC.GCAA.G.A....CCATT..CT.TAAGG.G...A....T	127	43.3	0	...G–.......–..–GA... CT.T...–TCA––.G.T..––T–...C	60.4
T8A	YP_001739778	*Thermotoga* sp. RQ2	...TCGA.T.AAAC.GCAA.G.A....CCATT..CT.TAAGG.G...A....T	127	43.3	0	...G–.......–..–GA...CT.T...–TCA––.G.T..––T–...C	60.4
T8B	YP_001739648	*Thermotoga* sp. RQ2	...TCGA.C.AAGT–GCAA.G.A....CCATT..CT.TAAGG.G...A....T	127	41.5	0	...G–....A..–.C–AT...CTGT...GT..–––G.T..––T–...C	54.5
T9A	YP_001245146	*Thermotoga petrophila* RKU-1	...TCGA.C.AAGT–GCAA.G.A....CCATT..CT.TAAGG.G...A....T	127	41.5	0	...G–.......–.C–AT...CTGT...GT..–––G.T..––T–...C	56.8

^1^ Distance (bp) to the transposase gene start (left) or stop (right) codons. Palindromic sequences are underlined. Identity as percentage (%).

**Fig 3:**
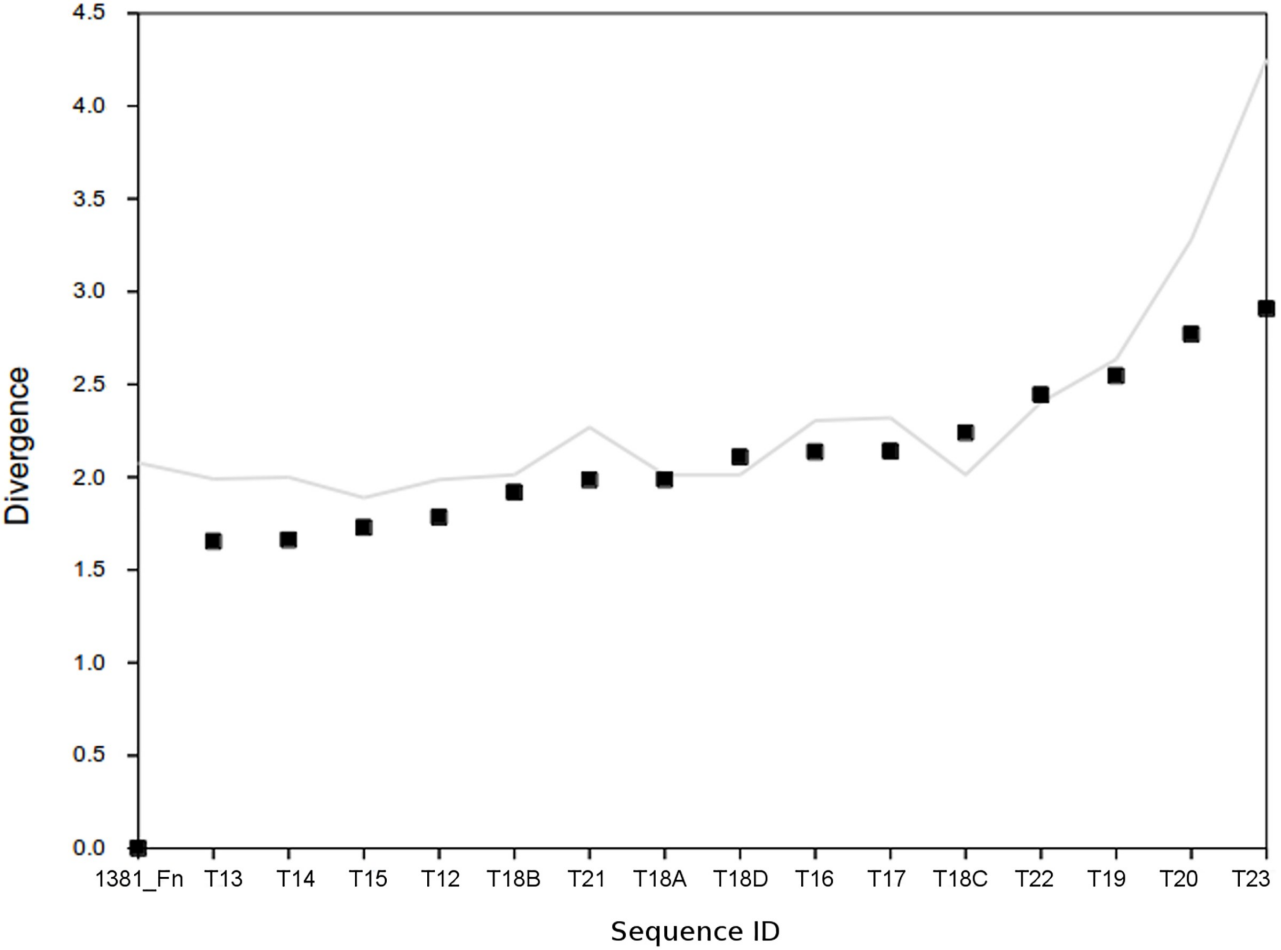
Ranking plot of K-L Divergence estimations for *F*. *nododum* and related gene sequences within the IS605_I transposase family. Squares represent pairwise divergence estimates between transposase genes; the line indicates the divergence estimates for transposase to whole genome comparisons. Identification of transposases follows the IDs used in the dendrogram for these sequences (Fig 1).

The K-L divergence estimator (Fig 3) also confirmed the proximity of *F*. *nodosum* and *Caldicellulosiruptor* transposases within IS605_I subgroup. An increase in divergence estimations was in agreement to phylogenetic distance (Fig 1). The other related transposase genes from other phyla showed slightly increasing divergence (Fig 3). The results from different methods confirmed a close relationship between *F*. *nodosum* IS605_I transposase and members of the Firmicutes and other phyla rather that to the closest Thermotogae sequences.

### ISL3 transposases

**Fig 4:**
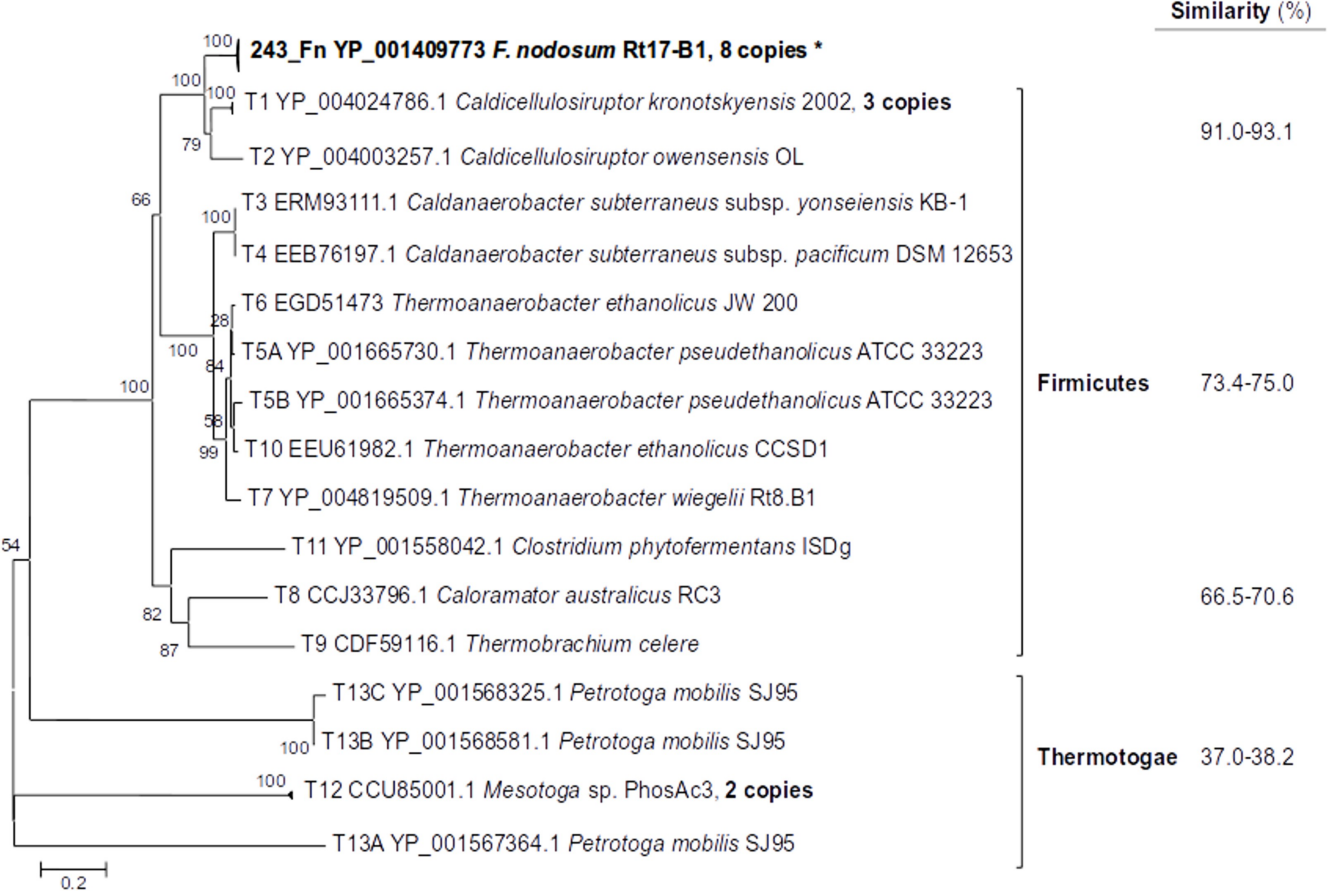
Phylogenetic relationship among *Fervidobacterium nodosum* and related gene sequences of the ISL3 transposase family and their closest relatives within the Phylum Thermotogae. The percentage of similarity related to the *F*. *nodosum* amino acidic sequence is shown on the right column.

Eight copies of the transposase genes detected in *F*. *nodosum* genome correspond to the ISL3 family (Table 1). The phylogenetic analysis showed that these sequences clustered together with transposases present in *Caldicellulosiruptor* (Firmicutes; 91–93% amino acid similarity) (Fig 4). Other Firmicutes, such as members of the genera *Caldanaerobacter* and *Thermoanaerobacter* also presented related sequences (73–75% amino acid similarity). Transposase genes from other Firmicutes genera, such as *Clostridium*, *Caloramator* and *Thermobrachium*, showed a bit lower similarity (66–71%). The closest sequences within the Phylum Thermotogae represented divergent clusters and much lower similarity to *F*. *nodosum* (37–38%) than the previous Firmicutes genera (Fig 4).

**Fig 5:**
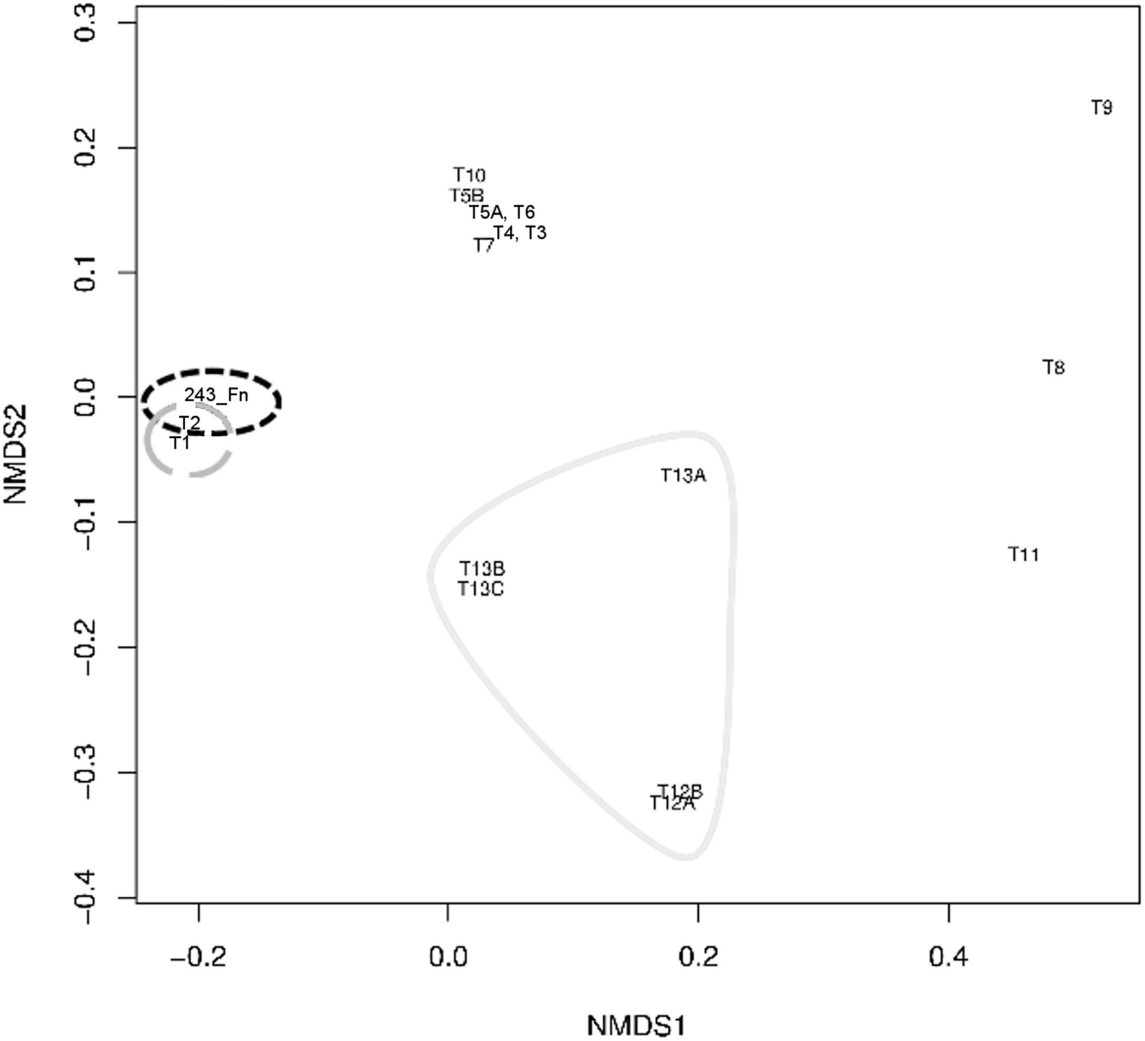
Non-metric MultiDimensional Scaling (NMDS) analysis of tetranucleotide frequencies for *F*. *nodosum* and related gene sequences of the ISL3 transposase family. Identification of transposases follows the IDs used in the dendrogram for these sequences (Fig 4). Dashed black, *F*. *Nodosum*; dashed grey, *Caldicellulosiruptor* (Firmicutes); continuous grey, other Thermotogae.

The clustering organization observed in Fig 4 was in agreement to their distributions on NMDS analysis of tetranucleotide frequencies (Fig 5). Thus, the major lineages differentiated on the phylogenetic dendrogram were also differentiated in the NMDS plot (Fig 5). *F*. *nodosum* and *Caldicellulosiruptor* sequences represented a single cluster, clearly differentiated to the other related sequences with decreasing similarity.

The IR sequences surrounding the ISL3 transposase genes in *F*. *nodosum* were practically identical among them (Table 3). Only minor differences (89–92% identity) were observed with their corresponding sequences in *Caldicellulosiruptor* (Table 3). Other Firmicutes ISL3 transposase genes showed close but slightly more distant IR sequences (75–86% identity) which paralleled to their increasing phylogenetic distance (Fig 4). The closest Thermotogae ISL3 transposase sequences were distant from those in *F*. *nodosum* with identities below 35% which also corresponded to the divergent phylogeny shown for this group (Fig 4).

**Table 3:** Repetitive flanking sequences detected in *F*. *nodosum* and related sequences within the ISL3 transposase family. The inverted repeats (IR) upstream (left end) and down-stream (right end) and their percentage of identity with respect to *F*. *nodosum* sequences are presented. Identification numbers correspond to those used in the figures for this transposase group.

ID	Accession No.	Organism	Left end^1^	Distance^2^	Identity^2^	Distance^2^	Right end	Identity^2^
243_fn	YP_001409773	*Fervidobacterium nodosum* RT17-B1	GGCTCTAT–AA–––T–AAATA–––TTGGGGTGGGTA–AA––AAG–GA–––––AAAAA–––––––TATAGCACTTATTCTGAATGCC	65		129	GGCATTCAGAA–––––––––TA––––––––––––––––––––––––––––––CCCACCCCA–C––TACTTGA–––––CAAAGAGCC	
343_Fn	YP_001409873	Fervidobacterium nodosum RT17-B1	........–..–––.–.....–––............–..––...–..–––––.....–––––––......................	65	100.0	129	...........–––––––––..––––––––––––––––––––––––––––––.........–.––.......–––––.........	100.0
999_Fn	YP_001410529	Fervidobacterium nodosum RT17-B1	........–..–––.–.....–––............–..––...–..–––––.....–––––––......................	65	100.0	129	...........–––––––––..––––––––––––––––––––––––––––––.........–.––.......–––––.........	100.0
1160_Fn	YP_001410690	Fervidobacterium nodosum RT17-B1	........–..–––.–.....–––............–..––...–..–––––.....–––––––......................	65	100.0	129	...........–––––––––..––––––––––––––––––––––––––––––.........–.––.......–––––.........	100.0
1322_Fn	YP_001410852	*Fervidobacterium nodosum* RT17-B1	........–..–––.–.....–––............–..––...–..–––––.....––––––––......................	65	100.0	129	...........–––––––––..––––––––––––––––––––––––––––––.........–.––.......–––––.........	100.0
1405_Fn	YP_001410935	Fervidobacterium nodosum RT17-B1	........–..–––.–.....–––........T...–..––...–..––––––.....–––––––......................	65	98.3	129	...........–––––––––..––––––––––––––––––––––––––––––.........–.––.......–––––.........	100.0
1408_Fn	YP_001410938	Fervidobacterium nodosum RT17-B1	........–..–––.–.....–––............–..––...–..–––––.....–––––––......................	65	100.0	129	...........–––––––––..––––––––––––––––––––––––––––––.........–.––.......–––––.........	100.0
1532_Fn	YP_001411062	Fervidobacterium nodosum RT17-B1	........–..–––.–.....–––............–..––...–..–––––.....–––––––......................	65	100.0	129	...........–––––––––..––––––––––––––––––––––––––––––.........–.––.......–––––.........	100.0
T2	YP_004003257	*Caldicellulosiruptor owensensis* OL	........–..–––.–.....–––.........A..G.TAG––.–..–––––.....–––––––......................	64	89.2	126	...........–––––––––..––––––––––––––––––––––––––––––.........A.––.–.....–––––......A..	92.5
T1A	YP_004024786	*Caldicellulosiruptor kronotskiensis* 2002	........–..–––.–.....–––.........–..–.––G..AA..–––––.....–––––––......................	65	92.1	128	..........G–––––––––..––––––––––––––––––––––––––––––.........A.––.–.....–––––.........	92.5
T1B	YP_004023466	*Caldicellulosiruptor kronotskiensis* 2002	........–..–––.–.....–––.........–..–.––G..AA..–––––.....–––––––......................	66	92.1	128	..........G–––––––––..––––––––––––––––––––––––––––––.........A.––.–.....–––––.........	92.5
T1C	YP_004022990	*Caldicellulosiruptor kronotskiensis* 2002	........–..–––.–.....–––.........–..–.––G..AA..–––––.....–––––––......................	65	92.1	128	...........–––––––––..––––––––––––––––––––––––––––––T........A.––.–.....–––––.........	92.5
T3	ERM93111	*Caldanaerobacter subterraneus* subsp. *yonseiensis* KB-1	..T.....–..–––.–....–––G..........G.A..AG..–ATCT–ATA.....–––––––G.G...................	53	76.0	nd^3^	...........AAA–––––A..––––––––––––––––––––––––––––––.........A.––..–....–––––.........	86.3
T4	EEB76197	*Caldanaerobacter subterraneus* subsp. *pacificus* DSM 12653	..T.....–..–––.–....–––G..........G.A..AG..–ATCT–ATA.....–––––––G.G...................	53	76.0	nd^3^	...........AAA–––––A..––––––––––––––––––––––––––––––.........A.––..–....–––––.........	86.3
T7	YP_004819509	*Thermoanaerobacter wiegelii* Rt8.B1	..T.....–..–––.–....–––G........AA..A..AG––.ATC–––TA.....–––––––C.G...................	49	75.3	152	...........AAA–––––A..––––––––––––––––––––––––––––––.........A.––..–....–––––.........	86.3
T5A	YP_001665730	*Thermoanaerobacter pseudethanolicus* ATCC 33223	..T.....–..–––.–....–––G..........G.A..AG..–ATCT–AT–.....–––––––G.G...................	52	77.1	164	...........AAA–––––A..––––––––––––––––––––––––––––––.........A.––..–....–––––.........	86.3
T6	EGD51473	*Thermoanaerobacter ethanolicus* JW200	..T.....–..–––.–....–––G........TAGGGG.–G..AA..––AT–.....–––––––..G...................	50	77.9	153	...........AAAA––––A..––––––––––––––––––––––––––––––.........A.––..–....–––––......A..	82.2
T10	EEU61982	*Thermoanaerobacter ethanolicus* CCSD1	..T.....–..–––.–....–––G..........G.A..AG..–ATCT–ATA.....–––––––G.G...................	52	76.0			
T5B	YP_001665374	*Thermoanaerobacter pseudethanolicus* ATCC 33223	..T.....–..–––.–....–––G..........G.A..AG..–ATCT–ATA.....–––––––G.G...................	52	76.0			
T13B	YP_001568581	*Petrotoga mobilis* SJ95	.....–––G..T––.C..–.–––––.–––––––––––––––..––TC––A––––––––––––––CTGC.–.–..–..–––––...A	273	34.8	8	AA..–––––..AAA–T–CGC.GTATTACCTTTTTCATCATCTCTTC–AGTCTATTCTAT..A.CA..T....–––––.........	29.4
T13C	YP_001568325	*Petrotoga mobilis* SJ95	...–.–T.G..AGG.C..–.–––––C–––––––––––––––...–T–––AT–––––––––––––C–G–.–.–.––..––––...GG	nd^3^	31.8	8	AA..–––––..AAA–T–CTC.GTAATACCTTTTTCATCATCTCTTC–AGTCTATTCT–T..A.CA..T....–––––...G.....	29.4

^1^ Underlined characters indicate a putative transposase start codon.

^2^ Distance (bp) to the transposase gene start (left) or stop (right) codons. Identity as percentage (%).

^3^ Not determined, nd.

**Fig 6:**
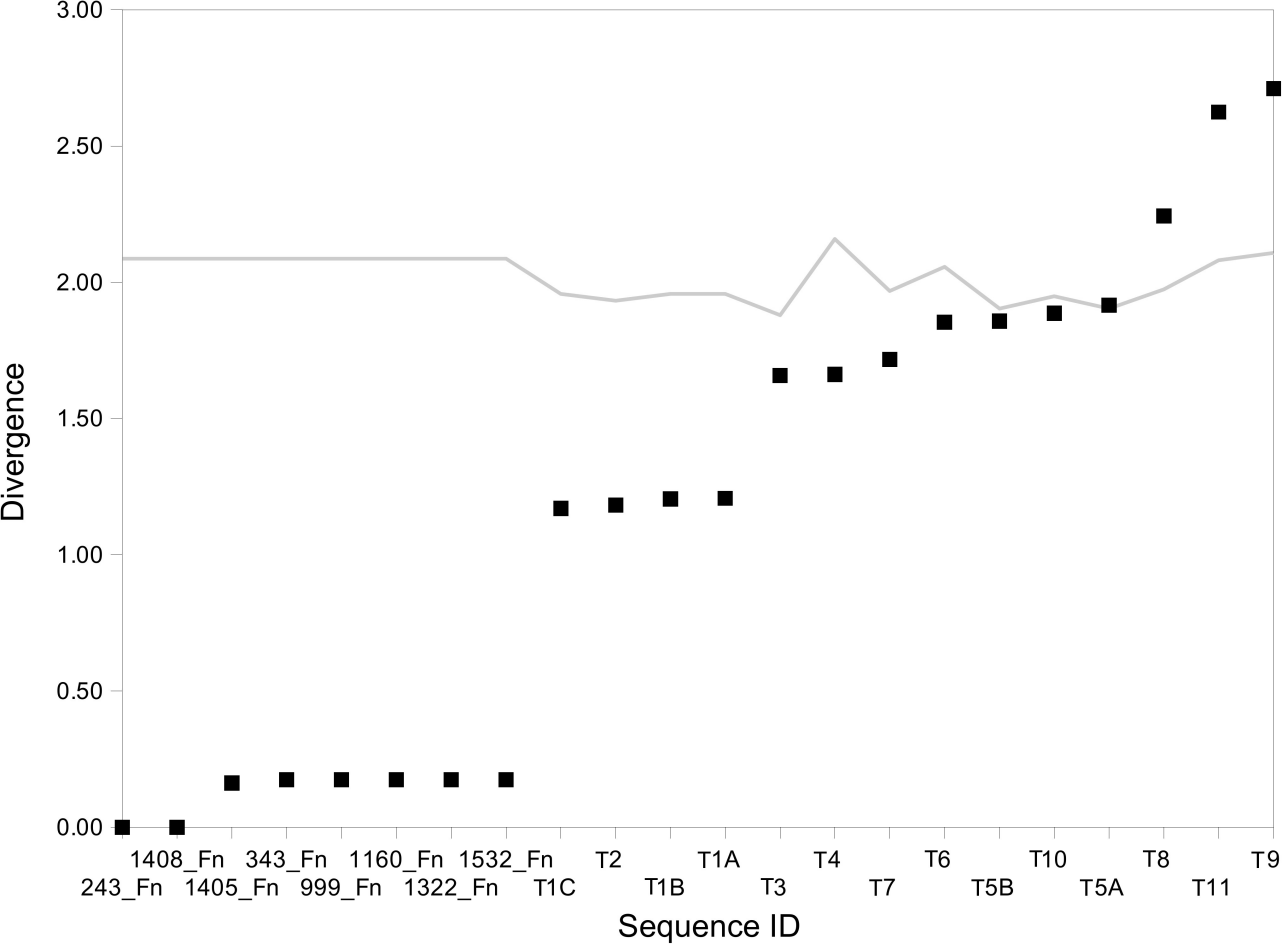
Ranking plot of K-L Divergence estimations for *F*. *nodosum* and related gene sequences within the ISL3 transposase family. Squares represent pairwise divergence estimates between transposase genes; the line indicates the divergence estimates for transposase to whole genome comparisons. Identification of transposases follows the IDs used in the dendrogram for these sequences (Fig 4).

Similarly, results from K-L divergence analyses (Fig 6) showed increasing divergence at increasing phylogenetic distance, in agreement to the results from Figs 4 and 5 and Table 3. The clusters observed at the phylogeny (Fig 4) were related to stepping up divergence and showed *Caldicellulosiruptor* as the genus with the lowest divergence from *F*. *nodosum* ISL3 transposase genes.

### Type C transposases

**Fig 7:**
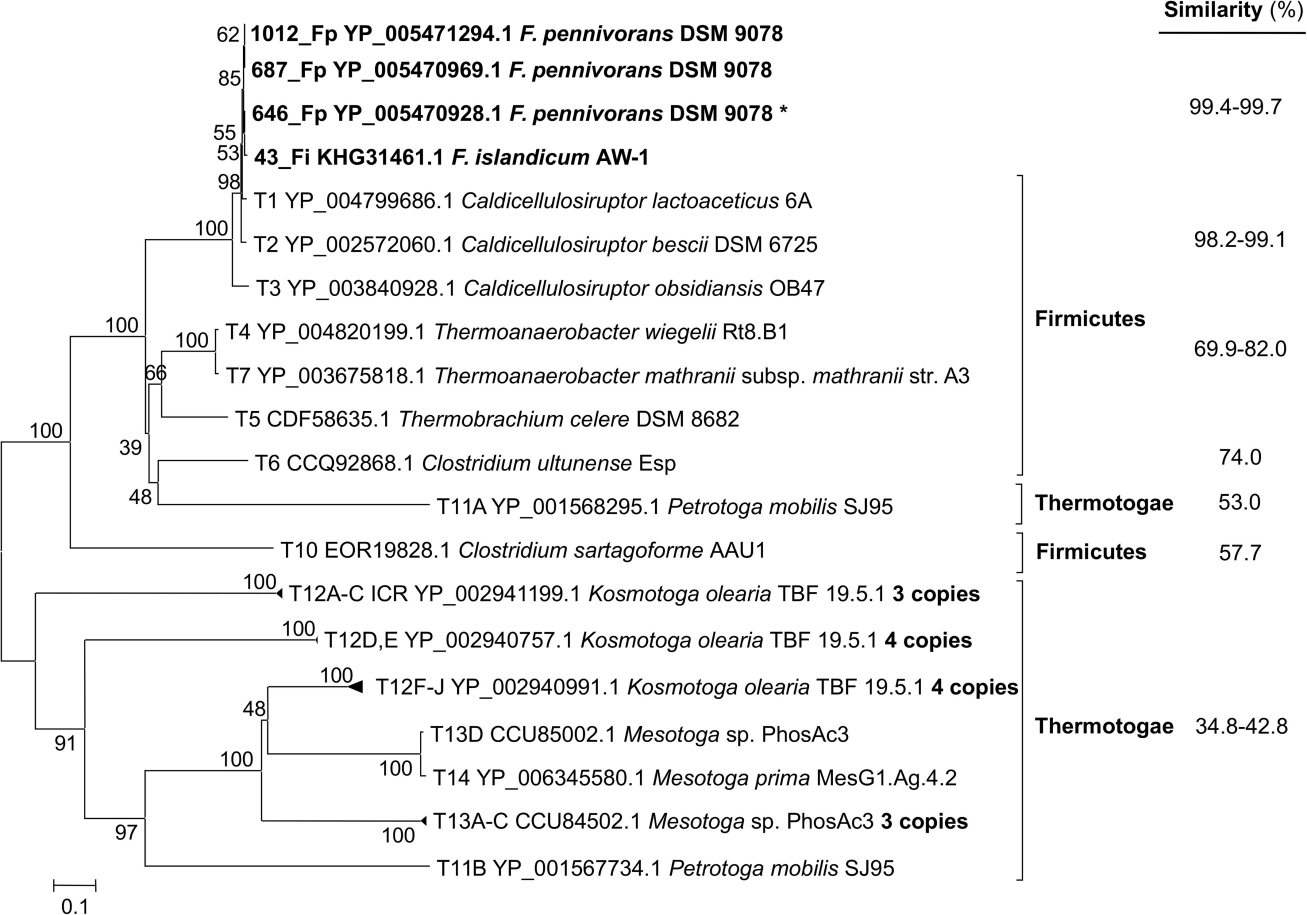
Phylogenetic relationship among *Fervidobacterium* and related gene sequences of the type C transposase group and their closest relatives within the Phylum Thermotogae. The percentage of similarity related to the *Fervidobacterium* amino acidic sequence is shown on the right column.

Three genes classified within the type C transposase group were found in *F*. *pennivorans* genome and one in *F*. *islandicum* genome. The closest sequences related to *Fervidobacterium* type C transposase genes belonged to the genus *Caldicellulosiruptor* (Firmicutes; 98–99% amino acid similarity) which formed a single clade together with the *Fervidobacterium* sequences (Fig 7). Other type C related transposases included transposases from Firmicutes such as *Thermoanaerobacter*, *Thermobrachium* and *Clostridium* (70–82% similarity). The closest Thermotogae transposase sequences belonged to *Petrotoga mobilis* (53% similarity). Other more distant sequences from Thermotogae forming an independent cluster were also detected but they showed levels of similarity below 43% (35–43%).

**Fig 8:**
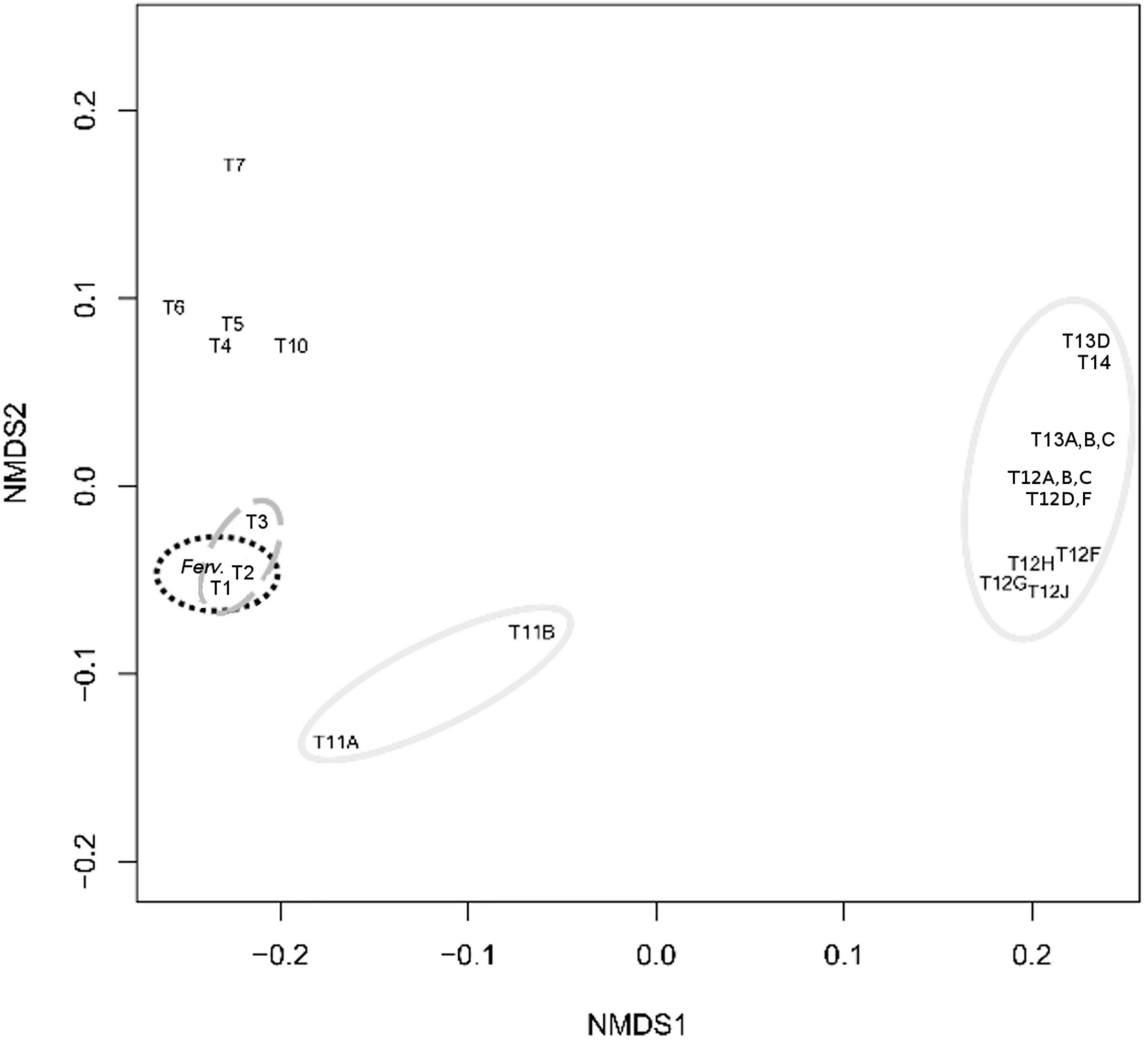
Non-metric MultiDimensional Scaling (NMDS) analysis of tetranucleotide frequencies for *Fervidobacterium* (*Ferv*.) and related sequences of the type C transposase group. Identification of transposases follows the IDs used in the dendrogram for these sequences (Fig 7). Dashed black, *Fervidobacterium* (*Ferv*.); dashed grey, *Caldicellulosiruptor* (Firmicutes); continuous grey, other Thermotogae.

NMDS analysis of tetranucleotide frequencies (Fig 8) differentiated the major phylogenetic clade formed by *Fervidobacterium* and *Caldicelulosiruptor* type C transposases. Additionally, other related phylogenetic clades observed within the Firmicutes clustered on the NMDS plot. Most Thermotogae sequences were clearly separated in a different and distant group (Fig 8).

*F*. *pennivorans* and *F*. *islandicum* showed identical IR sequences (Table 4). The IR sequence down-stream of *F*. *islandicum* type C transposase remained undetected. *Caldicellulosiruptor* type C transposases presented practically identical IR sequences (97–100% identity) than *Fervidobacterium*. Other related Firmicutes showed 74–86% identity to *Fervidobacterium* type C IR sequences. For the different lineage of Thermotogae, the IR sequence identity was much lower than the related Firmicutes, down to 42–56% identity.

**Table 4:** Repetitive flanking sequences detected in *Fervidobacterium* and related sequences of the type C transposase group. The inverted repeats (IR) upstream (left end) and down-stream (right end) and their percentage of identity with respect to *Fervidobacterium* sequences are presented. Identification numbers correspond to those used in the figures for this transposase group.

ID	Accession No.	Organism	Left end	Distance^1^	Identity^1^	Distance^1^	Right end	Identity^1^
646_Fp	YP_005470928	*Fervidobacterium pennivorans* DSM 9078	–––––––TGTTAATGATAAAATA–––AAAATGT–ACTGAAAGTGGAATATAAGGATGTACAAAAT–TGTAC–––	41		0	–––––––––––––GTTTATTTTTTGTACATTTTTATTTTCGAT–TTTTTGTTCATTTTGA–TATTGACATTTACA–	
1012_Fp	YP_005471294	*Fervidobacterium pennivorans* DSM 9078	–––––––................–––.......–...............................–.....–––	41	100.0	0	–––––––––––––..............................–................–..............–	100.0
687_Fp	YP_005470969	*Fervidobacterium pennivorans* DSM 9078	–––––––................–––.......–...............................–.....–––	41	100.0	0	–––––––––––––..............................–................–..............–	100.0
43_Fi	KHG31461	*Fervidobacterium islandicum* AW-1	–––––––................–––.......–...............................–.....–––	41	100.0	nd^2^	––––CAGAAATTTTGCGTCC.GG.T..TGC.G..G.GC.TT..–....–...–....AT.T....–T.–.AG–..–	50.8
T1	YP_004799686	*Caldecellulosiruptor lactoaceticus* 6A	–––––––................–––.......–...............................–.....–––	41	100.0	0	–––––––––––––..............................–................–..............–	100.0
T2	YP_002572060	Caldecellulosiruptor lactoaceticus 6A	–––––––G...............–––.......–..................T............–.....–––	41	96.6	0	–––––––––––––..............................–................–..............–	100.0
T3	YP_003840928	Caldecellulosiruptor lactoaceticus 6A	–––––––....G...........–––.......–...................A...........–.....–––	41	96.6	0	–––––––––––––..............................–................–..............–	100.0
T7	YP_003675818	*Thermoanaerobacter matharanii* A3	––––––C...A...A........–––.G.....–..GA.........A....AA.......G.T.–.....–––	82	81.7	nd^2^	–––––––––––––..AC............AAA....A...C.C–.......A..AAAAT.–.......T......G	73.7
T6	CCQ92868	*Clostridium ultunense* Esp	––––––––..A...........T–––.G.....–..AATT.A.....A.....A..........G–..––––––	36	80.3	nd^2^	––––––––––––––––––––..........AA........C.C–......A.....C.T.–.............––	86.5
T12B	YP_002941199	*Kosmotoga olearia* TBF 19.5.1	GTGTCGACA.CG..T........GTA....C..CGG.C...AAT..GC.A..AC..CGGT..G.AT..AGTAGT	38	42.4	nd^2^	ACTACGCAAATCT.A.CGA.GAAAC...GCAA..T.G......G.....A.G.T....TG––G.C..TG..G...–	55.7
T12A	YP_002940250	*Kosmotoga olearia* TBF 19.5.1	GTGTCGACA.CG..T........GTA....C..CGG.C...AAT..GC.A..AC..CGGT..G.AT..AGTAGT	38	42.4	nd^2^	ACTACGCAAATCT.AGCGA.GAAAT...GCAA..T.GA.....G.....T.G......TG––G....TG..G...–	55.7
T12C	YP_002941123	*Kosmotoga olearia* TBF 19.5.1	GTGTCGACA.CG..T........GTA....C..CGG.C...AAT..GC.A..AC..CGGT..G.AT..AGTAGT	38	42.4	nd^2^	ACTACGCAAATCT.A.CGA.GAAAC...GCAA..T.G......G.....A.G.T....TG––G.C..TG..G...–	55.7

^1^ Distance (bp) to the transposase gene start (left) or stop (right) codons. Identity as percentage (%).

^2^ Not determined, nd.

**Fig 9:**
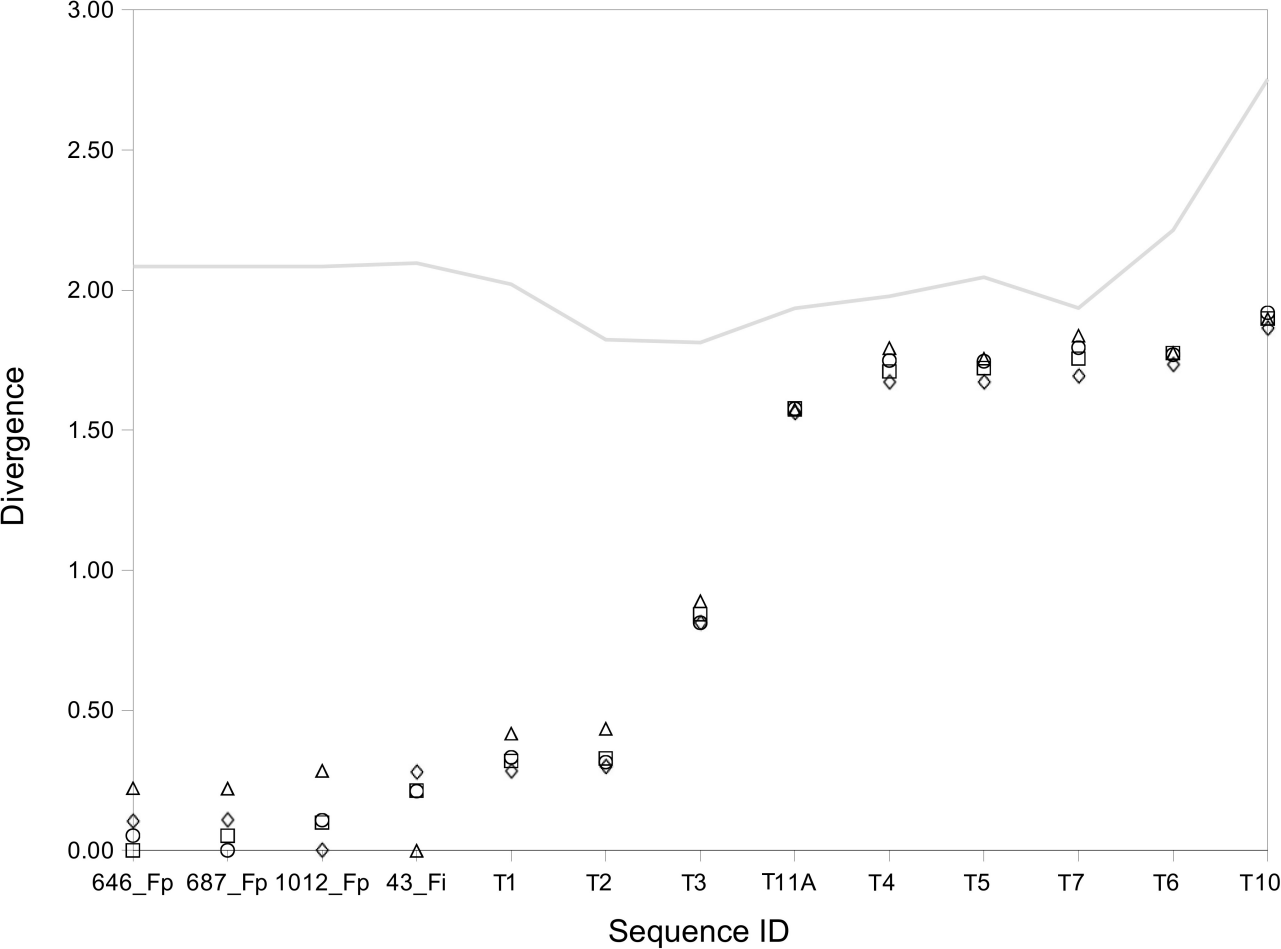
Ranking plot of K-L Divergence estimations for *Fervidobacterium* and related gene sequences of the type C transposase group. Symbols represent pairwise divergence estimates between transposase genes; the line indicates the divergence estimates for transposase to whole genome comparisons. Divergence estimates were calculated with respect to sequences 43_Fi (triangules), 646_Fp (squares), 687_Fp (circles) and 1012_Fp (diamonds). Identification of transposases follows the IDs used in the dendrogram for these sequences (Fig 7).

K-L divergence analyses of tetranucleotide frequencies also confirmed the proximity of *Caldicellulosiruptor* type C sequences to *Fervidobacterium* (Fig 9). Low divergence estimations were observed between *Fervidobacterium* and *Caldicellulosiruptor* type C transposase genes. Other Firmicutes and Thermotogae presented much higher K-L Divergence values.

### Type D transposases

**Fig 10:**
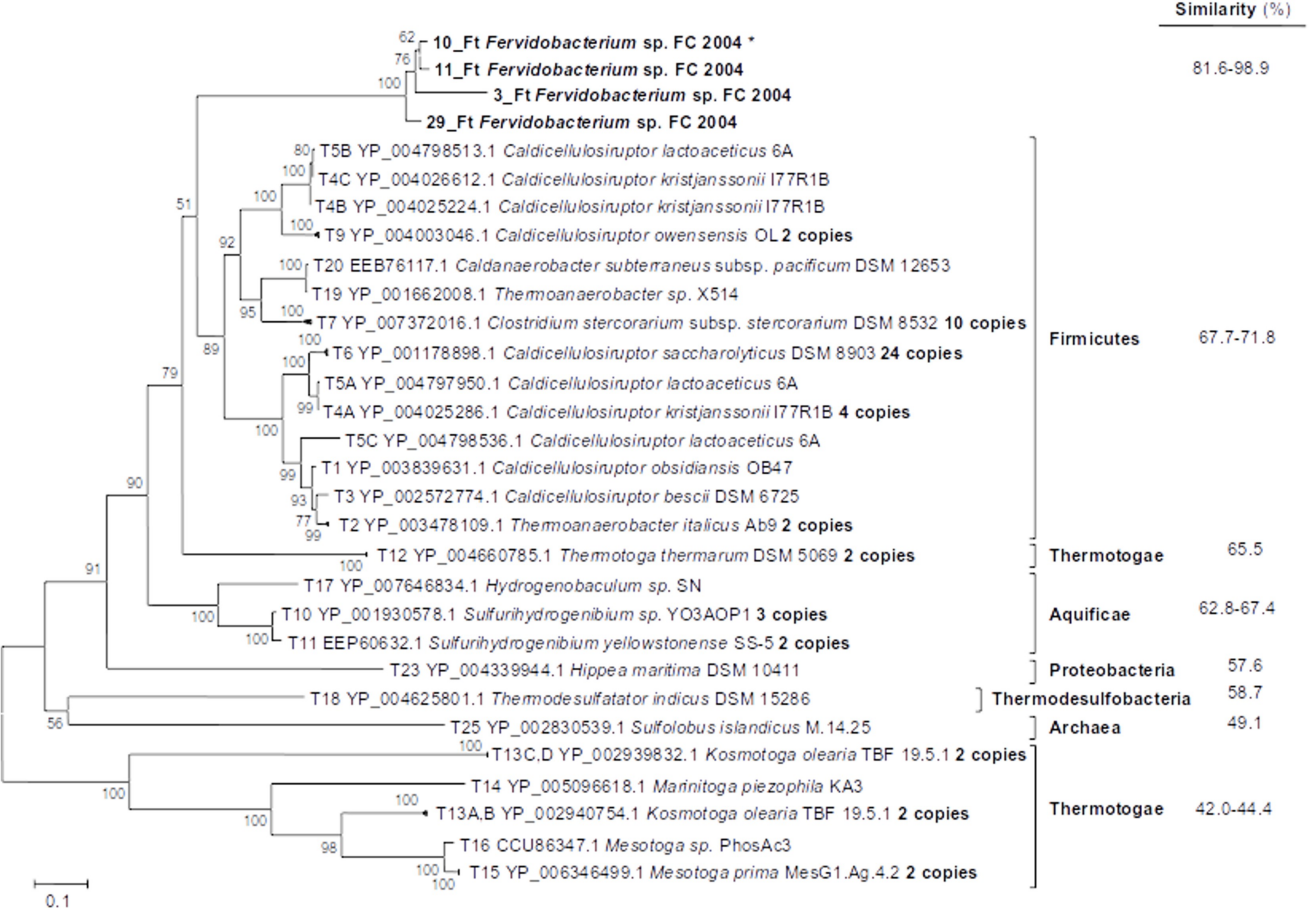
Phylogenetic relationship among *Fervidobacterium* sp. FC2004 and related gene sequences of the type D transposase group and their closest relatives within the Phylum Thermotogae. The percentage of similarity related to the *Fervidobacterium* amino acidic sequence is shown on the right column.

Transposase type D was only detected in *Fervidobacterium* sp. strain FC2004 with five copies found in its genome. These *Fervidobacterium* transposase genes presented as closest relatives genes from Firmicutes (68–72% similarity; Fig 10), specifically from the genera *Caldicellulosiruptor* and some sequences from *Caldanaerobacter*, *Thermoanaerobacter* and *Clostridium* which formed a single cluster to the *Fervidobacterium* type D transposases. A relatively close transposase gene was found in *Thermotoga thermarum* but it formed a divergent clade and presented lower similarity (65% similarity) that those Firmicutes genes. Other related genes from the Thermotogae presented much lower similarities (42–44%) and represented a divergent clade. Transposase genes forming related lineages with decreasing similarity to *Fervidobacterium* type D transposases were detected in Aquificae (63–67%), Proteobacteria (58%), Thermodesulfobacteria (59%) and Archaea (49%) (Fig 10).

**Fig 11:**
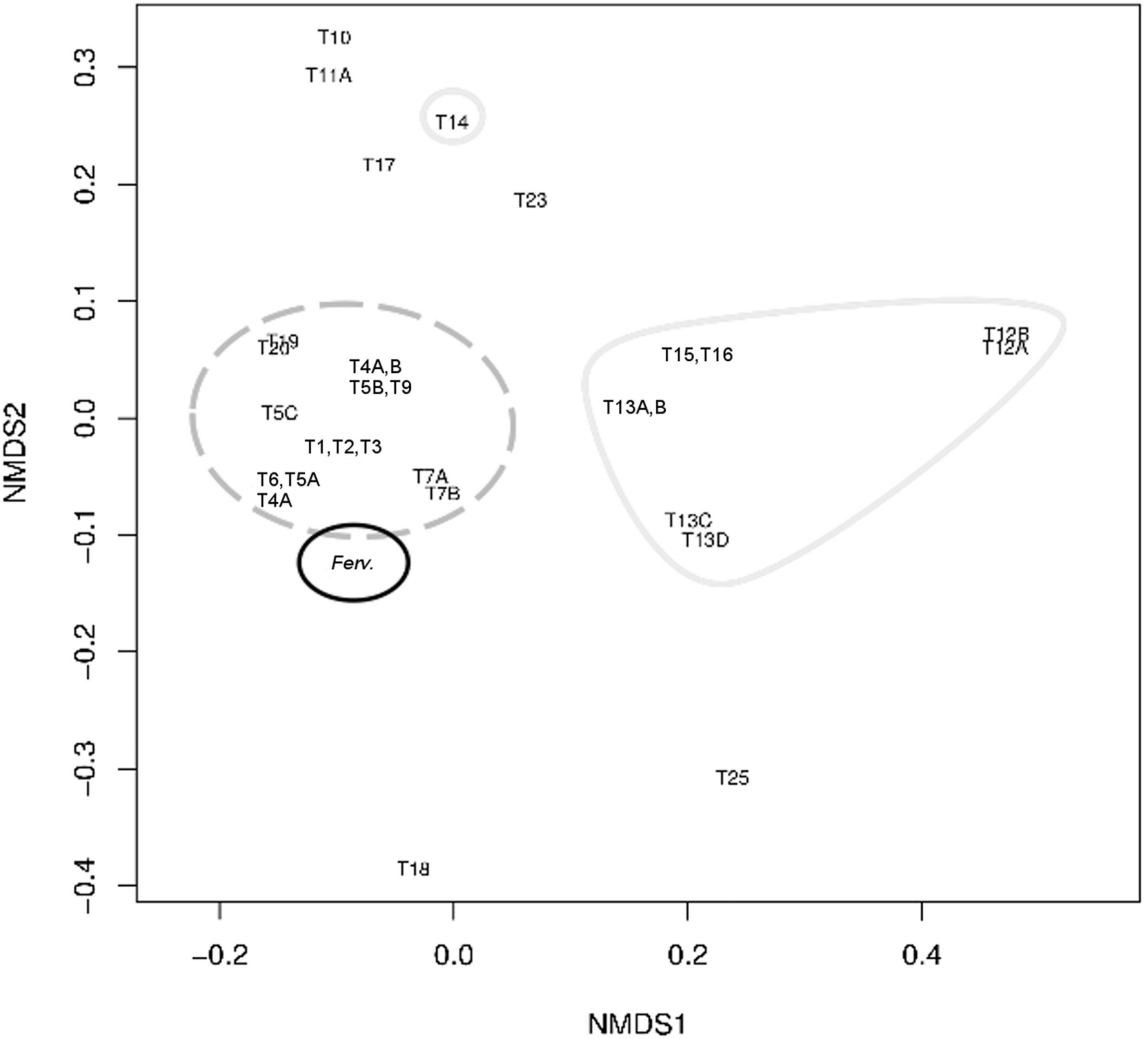
Non-metric MultiDimensional Scaling (NMDS) analysis of tetranucleotide frequencies for *Fervidobacterium* sp. FC2004 (*Ferv*.) and related sequences of the type D transposase group. Identification of transposases follows the IDs used in the dendrogram for these sequences (Fig 10). Continuous black, *Fervidobacterium* sp. FC2004 (*Ferv*.); dashed grey, Firmicutes; continuous grey, other Thermotogae.

A similar pattern to that observed from the phylogeny was observed through NMDS analysis of tetranucleotide frequencies (Fig 11). The *Fervidobacterium* type D transposase genes clustered closed to the most related Firmicutes genes and clearly differentiated of more distant transposase genes from other taxonomic groups, including Thermotogae.

Palindromic IR sequences were detected surrounding the transposase type D genes (Table 5). The IRs within the *Fervidobacterium* FC2004 genome were highly conserved (95–100% identity). Sequence identity between the IRs of *Fervidobacterium* and some *Caldicellulosiruptor* species were 59% and 67% up- and down-stream, respectively. In other Firmicutes (*Caldicellulosiruptor*, *Thermoanaerobacter*, *Clostridium*) identities between 50% to 69% were observed revealing proximity between IR sequences from *Fervidobacterium* and some Firmicutes. The closest Thermotogae transposase genes to these type D transposases presented IR nucleotide identities between 30% to 43% in agreement to a much higher phylogenetic distance than the pointed Firmicutes to *Fervidobacterium* type D transposase genes.

**Table 5:** Repetitive flanking sequences detected in *Fervidobacterium* sp. FC2004 and related sequences of the type D transposase group. The palindromic inverted repeats upstream (left end) and down-stream (right end) and their percentage of identity with respect to *Fervidobacterium* sp. FC2004 sequences are presented. Identification numbers correspond to those used in the figures for this transposase group.

ID	Accession No.	Organism	Left end	Distance^1^	Identity^1^	Distance^1^	Right end	Identity^1^
10_Ft	LWAF01000000	*Fervidobacterium thailandense* FC 2004	GGGAGTACAATAAATTTTGTGTCTGGAAAATTTTAGCTTGCTAA	40		71	GTGTTTGTTCTCTTTTCTTCGGACACAAAATTCTTGGACA–GTCTC	
11_Ft	LWAF01000000	*Fervidobacterium thailandense* FC 2004	...............................C............	40	97.7	70	...G.................................T..–.....	95.5
29_Ft	LWAF01000000	*Fervidobacterium thailandense* FC 2004	..........G.................................	40	97.7	nd^2^	...G.................................T..–.....	95.5
3_Ft	LWAF01000000	*Fervidobacterium thailandense* FC 2004	............................................	40	100.0	71	...G...............A....................–.....	95.5
T5B	YP_004798513	*Caldecellulosiruptor lactoaceticus* 6A	...T..TA..AT..AA.........A.GT..GA..AAA.CGC..	15	59.0	-33	CA...ACGCTA.–...ACC.A...............–...A.....	67.3
T4C	YP_004026612	*Caldecellulosiruptor kristjanssonii* I77R1B	...T..TA..AT..AA.........A.GT..GA..AAA.CGC..	15	59.0	-33	CA...ACGCTA.–...ACC.A...............–...A.....	67.3
T4B	YP_004025224	*Caldecellulosiruptor kristjanssonii* I77R1B	...T..TA..AT..AA.........A.GT..GA..AAA.CGC..	15	59.0	-33	CA...ACGCTA.–...ACC.A...............–...A.....	67.3
T9A	YP_004003046	*Caldecellulosiruptor owensensis* OL	...T..TA..AT..AG.........AT.T..AA..AAA.CAC..	50	59.0	-33	CA...ACGCTA.–...ACC.A...............–...A.....	67.3
T9B	YP_004003334	*Caldicellulosiruptor owensensis* OL	...T..TA..AT..AG.........AT.T..AA..AAA.CAC..	51	59.0	-33	CA...ACGCTA.–...ACC.A...............–...A.....	67.3
T20	EEB76117	*Caldanaerobacter subterraneus* DSM 12653	..AT..TA..AT..AA.........ATGT..AA.GAAA.TG...	332	54.5	-33	CA...ACG.TA.–...AAC.A...............–...A.....	69.5
T19	YP_001662008	*Thermoanaerobacter* sp. X514	..AT..TA..AT..AA.........ATGT..AA.GAAA.TG...	333	54.5	-33	CA...ACG.TA.–...AAC.A...............–...A.....	69.5
T7A	YP_007372016	*Clostridium stercorarium* DSM 8532	...T..TA..AT..AA..........TGT..AA..AAA.CAC..	15	59.0	-33	CA...ACG.TA.–...AA..A...............–...AC....	69.5
T7B	YP_007373409	*Clostridium stercorarium* DSM 8532	...T..TA..AT..AA..........TGT..AA..AAA.CAC..	15	59.0	16	CA...ACG.TA.–...AA..A...............–...AC....	69.5
T6A	YP_001179002	*Caldicellulosiruptor saccharolyticus* DSM 8903	......TA..AT..AA.......CACTGT..AA.TAG.GTTG..	27	52.2	-33	AATA.ACGCTAT–GAAA.GGAA..............–...A.....	52.1
T6B	YP_001178898	*Caldicellulosiruptor saccharolyticus* DSM 8903	......TA..AT..AA.......CACTGT..AA.TAG.GTTG..	27	52.2	-33	AATA.ACGCTAT–GAAA.GGAA..............–...A.....	52.1
T6C	YP_001181500	*Caldicellulosiruptor saccharolyticus* DSM 8903	.AA...TA..AT..AA.......CATTGT.GAA.TAG.ATTG..	78	45.4	-12	AATA.ACGCTAT.GA.G.GGAA..............–...A.....	56.5
T5A	YP_004797950	*Caldicellulosiruptor lactoaceticus* 6A	......TA..AT..AA.......CACTGT..AA.TAG.ATTG..	26	52.2	-33	AATA.GCGCTAT–GAAA.GGAA..............–...A.....	52.1
T4A	YP_004025286	*Caldicellulosiruptor kristjanssonii* I77R1B	......TA..AT..AA.......CACTGT..AA.TAG.ATTG..	26	52.2	-33	AATA.GCGCTAT–GAAA.GGAA..............–...A.....	52.1
T5C	YP_004798536	*Caldicellulosiruptor lactoaceticus* 6A	......TA..AT..AA........ACTGT..AA.TAG.ATTG.G	25	52.2	-33	AAC..ACG.TAT–AAAT.GGAA..............–...A.....	56.5
T1	YP_003839631	*Caldicellulosiruptor obsidiansis* OB47	......TA..AT..AA.......CACTGT..AA.TAG.ATTG..	26	52.2	-33	AAT..ACG.TAT–AAAT.GGAA..............–...A.....	56.5
T3	YP_002572774	*Caldicellulosiruptor bescii* DSM 6725	......TA..AT..AA.......CACTGT..AA.TAG.ATTG..	26	52.2	-33	AAC..GCG.TAT–AAAT.GGAA..............–...A.....	56.5
T2A	YP_003478109	*Thermoanaerobacter italicus* Ab9	......TA..AT..AA.......CACTGT..AA.TAGAATTG..	26	50.0	-33	AAC..ACG.TAT–AAGT.GGAA..............–...A.....	56.5
T2B	YP_003476860	*Thermoanaerobacter italicus* Ab9	......TA..AT..AA.......CACTGT..AA.TAGAATTG..	26	50.0	-33	AAC..ACG.TAT–AAGT.GGAA..............–...A.....	56.5
T12	YP_004660785	*Thermotoga thermarum* DSM 5069	......T.G.AT..............TGGTAAGATTA...TAC.	17	59.0	-35	AAC.G–AGGTAT–...G.C.A............ACC–CT.A.....	52.1
T17	YP_007646834	*Hydrogenobaculum* sp. SN	......TA..AT.T..A.......TA..CT.AAACA...TA.C.	20	59.0	-33	AACAGGAGAT.T–.G.TA.GA...........A...–...A.....	56.5
T10	YP_001930578	*Sulfurihydrogenibium* sp. YO3AOP1	......TA..AT...G........AA.TT.AA..TATAA.GAGG	7	54.5	-33	AATAAAAAGT.T–.CAA.C.A...........A...–.T.A.....	54.3
T23	YP_004339944	*Hippea maritima* DSM 10411	....C.TA..AT.T..........CCCCC.CAAACCTACTAA..	69	47.7	7	––––..TGGTATGA.GAGAGA.........A.TAG.–GG.T.....	43.4
T18	YP_004625801	*Thermodesulfatator indicus* DSM 15286	.A.G.CGGG.A...........AA..GT..AA..TAAGAAACTC	19	47.7	4	AACAGGT.ATCAGC.AACCTTT..........TC.T–.T.G.C...	41.3
T25	YP_002830539	*Sulfolobus islandicus* M4.25	.AAGC.CGGGGCT.CCAGT.A.AGTTGTGG.AG.CAAAG.TG..	5	22.7	8	––––––––.TA.A.A.T...TT......CTA.ACCC–.GTA.C.C.	39.1
T13A	YP_002940754	*Kosmotoga olearia* TBF 19.5	...TC.GTT.GC..........AAACTCCC.CCG.AA.ATGAG.	29	43.1	nd^2^	TCT...CCGGAG...GGGGTTT.........ATG..–...C.C.CG	47.8
T13B	YP_002940481	*Kosmotoga olearia* TBF 19.5	...TC.GTT.GT..........AAACTCCC.CCG.AA.ATGAG.	29	43.1	nd^2^	TCT...CCGGAG...GGAGTTT.........ATG..–...T.C.CG	47.8
T15A	YP_006346499	*Mesotoga prima* MesG1.Ag.4.2	.A.TA.GT..AG..GA......AAACCCTC.AGA.TGGAAGAG.	19	36.3	7	ACCCA.TACAAAAAAGGGGTTT........AA–GA.–...GAA.C–	30.4
T15B	YP_006345816	*Mesotoga prima* MesG1.Ag.4.2	.A.TA.GT..AG..GA......AAACCCTC.AGA.TGGAAGAG.	19	36.3	7	ACCCA.TACAAAAAAGGGGTTT........AAAGA.–...GAA.C–	30.4

^1^ Distance (bp) to the transposase gene start (left) or stop (right) codons. Identity as percentage (%).

^2^ Not determined, nd.

**Fig 12:**
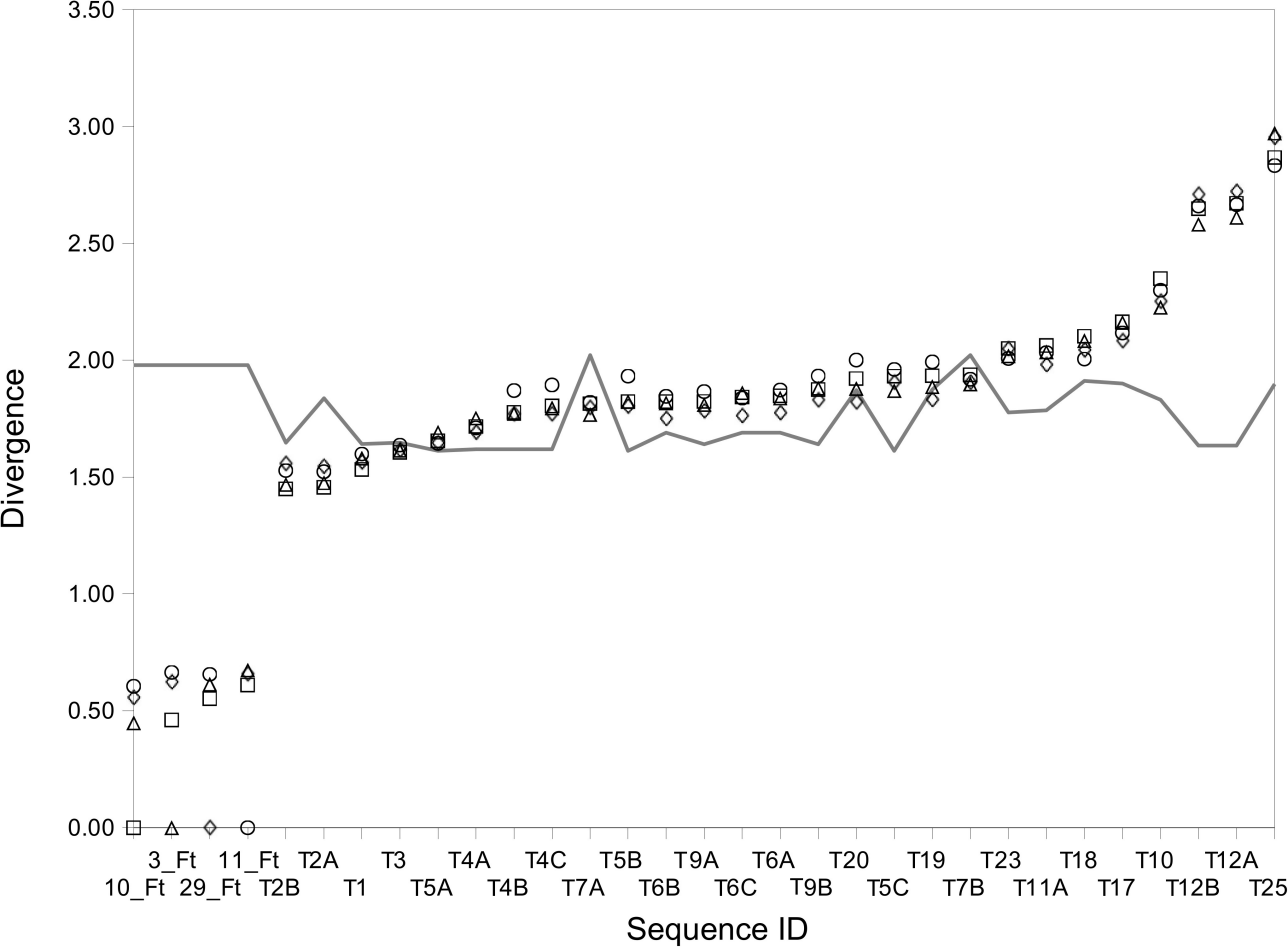
Ranking plot of K-L Divergence estimations for *Fervidobacterium* sp. FC2004 and related gene sequences of the type D transposase group. Symbols represent pairwise divergence estimates between transposase genes; the line indicates the divergence estimates for transposase to whole genome comparisons. Divergence estimates were calculared with respect to the sequences 3_Ft (triangules), 10_Ft (squares), 11_Ft (circles) and 19_Ft (diamonds). Identification of transposases follows the IDs used in the dendrogram for these sequences (Fig 10).

Gene sequences corresponding to type D transposases presented lowest K-L Divergence estimates when comparing *Fervidobacterium* and the Firmicutes indicated above. Increasing phylogenetic distance (Fig 10) resulted in a progressive increase of K-L Divergence estimates (Fig 12) suggesting parallel results between the different methods of analysis of HGT used in this study.

## Discussion

Transposases are highly related to the mobility of DNA within and likely between genomes [6, 7] which suggests that they represent a proxy to detect and analyze HGT events and its influence on bacterial evolution and genome plasticity. In this case, we approached the study of transposases in *Fervidobacterium* genomes. Using different methods to test for potential HGT events of these sequences, the results indicated the presence of a number of transposases reflecting HGT events.

Due to the generally observed IS sequence conservation over a broad range of prokaryotic taxa [9, 30] and their functional trait promoting their own duplication, transposase genes are likely to show relatively homogeneous sequences in comparison to other genes forming the pan genome of a taxon. Wagner [9] proposed that ISs may go periodically extinct in prokaryotic populations and become reintroduced through HGT events alleviating the potential negative long-term effects of their massive expansion in the prokaryote genomes. Nevertheless, ISs are suggested to represent evolutionary benefits in a short run which justifies their implication in species adaptation and genome plasticity [5, 6, 31]. Thus, the detection of transposase genes within a genome represents a snapshot of a prokaryote genome and provides indications of the potential genetic flow affecting the relatively recent history of specific taxa.

Using the variability range from a reference IS sequence within a *Fervidobacterium* genome and the closest relatives and clades within other Thermotogae allows to approach the distinction of those ISs putatively incorporated through interphylum HGT events. Thus, these phylogenetically distant HGT events are easily discriminated from potential vertical transferences of DNA within a taxon genomic context. The approach involved different strategies to discriminate IS sequences including phylogeny and tree topology, multidimensional analysis and divergence metrics on tetranucleotide frequencies, and comparative IR sequence conservation. These methods were mostly in agreement on suggesting HGT events of IS sequences between *Fervidobacterium* and other phyla. The use of transposase genes as target sequences and different approaches to detect interphylum HGT events has shown to represent valuable tools for HGT analyses.

Phylogeny showed clear clustering of similar sequences. Besides, multidimensional analyses (i.e., NMDS) of tetranucleotide frequencies also confirmed clustering of the distribution of transposase sequences from *Fervidobacterium* and related transposase genes. Divergence metrics of transposase genes confirmed increased values in parallel to increasing phylogenetic distance. In addition, the detection of IR sequences flanking those transposase genes is consistent with the idea of them being functional and likely their relatively recent incorporation [9] into these genomes even if the actual mechanism of transference is hardly understood.

This study clearly shows the relationship on gene sharing between *Fervidobacterium* genomes and other prokaryotic phyla. The most frequently detected interphylum HGT relationship was observed between *Fervidobacterium* and Firmicutes genomes. Specifically, *Caldicellulosiruptor* genomes shared multiple common ISs with *Fervidobacterium* genomes and some of them presented up to 99% amino acid similarity in their transposases and 100% IR nucleotide sequence identities. These results were compared to the closely detected IS sequence within each IS family and other Thermotogae (beyond the *Fervidobacterium* genus) which showed amino acid similarities and IR nucleotide sequence identities generally below 50%. These data confirmed the occurrence of clear interphylum HGT events between those two genera.

Other members of the Firmicutes (i.e., *Thermoanaerobacter*, *Caldanaerobacter*, *Caloramator*, *Thermobracchium* and *Clostridium* among others) also showed putative HGT events with the genus *Fervidobacterium*. As well, bacteria from other phyla (i.e., Aquificae, Synergistetes, Deinococcus-Thermus, Thermodesulfobacteria, Proteobacteria) and some Archaea were also detected as putatively involved in relatively recent HGT events with *Fervidobacterium*. Interestingly, most of these representatives presenting HGT relationship to *Fervidobacterium* were also thermophiles which confirmed the requirement of potentially sharing a habitat to make DNA exchanges possible [30].

## Conclusions

The diversity of transposase genes detected within the genus *Fervidobacterium* suggests that these sequences have a major contribution to the adaptation and evolution of these bacteria and their genomes to the environment. The actual implication of transposases, their role in HGT, the rate of HGT events and transposase duplication and exchange remain to be fully understood. This study shows the relevance of transposases on HGT using the *Fervidobacterium* genus as a model of study and shows that IS sequences can be used to detect the occurrence of HGT events between phylogenetically distant prokaryotes and to identify the taxa involved in potential exchanges of genomic DNA.
